# The Impact of Social Media Influencers on Children’s Dietary Behaviors

**DOI:** 10.3389/fpsyg.2019.02975

**Published:** 2020-01-10

**Authors:** Crystal R. Smit, Laura Buijs, Thabo J. van Woudenberg, Kirsten E. Bevelander, Moniek Buijzen

**Affiliations:** ^1^Behavioural Science Institute, Radboud University, Nijmegen, Netherlands; ^2^Radboud University Medical Center, Nijmegen, Netherlands; ^3^Erasmus School of Social and Behavioural Sciences, Erasmus University Rotterdam, Rotterdam, Netherlands

**Keywords:** children, food marketing, vlogs, social media influencers, sugar-sweetened beverages, energy-dense snacks, dietary behaviors

## Abstract

Over the past years vlogs rapidly have become an attractive platform for food industries, sponsoring social media influencers to promote their products. As with more traditional media, social media influencers predominantly promote unhealthy drinks and foods that are high in sugar, fat, and salt – consumption of which may increase the risk of overweight, obesity, and non-communicable diseases. The aim of the current Brief Research Report is to examine the impact of vlogs on children’s unhealthy dietary behaviors. Drawing on longitudinal survey data from 453 8- to 12-year-old children, we analyzed the longitudinal relations between children’s frequency of watching vlogs and their consumption of unhealthy beverages and snacks. Structural path modeling analyses of three waves of data with 1-year intervals showed that children’s self-reported frequency of watching vlogs influenced consumption of unhealthy beverages 2 years later. The analyses did not yield significant relations for Unhealthy Snacks Consumption. The strength of the observed longitudinal relation between children’s Frequency of Watching Vlogs and Consumption of unhealthy beverages was comparable to previous findings regarding more traditional types of food marketing.

## Introduction

In the debate about the childhood obesity crisis, food marketing in media is often named as one of the main causes for children’s unhealthy dietary behaviors (WHO, 2010; Boyland et al., 2016; Robinson et al., 2017). Research shows that, similar to traditional media such as television, “vlogs” (i.e., video weblogs) by social media influencers are a popular platform for advertisers to target young audiences with food marketing (Tan et al., 2018; Folkvord et al., 2019). This influencer marketing technique involves the promotion and selling of products or services through social media personalities (“influencers”) who have the capacity to affect the character of a brand (Hill et al., 2020). As is the case with food marketing in more traditional media, social media influencers predominantly promote drinks and foods that are high in sugar, fat, and salt (Coates et al., 2019; Folkvord et al., 2019). Consumption of these unhealthy products increases the risk of overweight, obesity, and non-communicable diseases (WHO, 2010).

Vlogs of social media influencers are an attractive platform for food industries to promote their products. Watching these vlogs has become a huge part of children’s daily media consumption, for many children even taking the place of watching television programs (Ofcom, 2017). Due to this shift in media activities, vlog-based advertising has become an important way for the food industry to reach the largest audiences of children. Moreover, while the traditional media environment has witnessed considerable restrictions of child-directed food in the past decades, sometimes even involving total bans of food advertising, the online video environment is still relatively unregulated. Thereby, vlogs are among the few media channels where beverages and foods can still be advertised without restrictions.

Food marketing by social media influencers is also of scholarly interest, because it raises new questions about the processes and effects of non-traditional advertising formats. Based on advertising processing models (Buijzen et al., 2010; Rozendaal et al., 2011), it can be expected that sponsoring of social media influencers to advertise products in vlogs is a particularly effective strategy to influence children. Importantly, the food promotion is often highly integrated with the vlog content, rendering it more difficult for children to recognize it as advertising than is the case with more traditional advertising, such as television commercials. With recognition of advertising being a first prerequisite to process advertising critically (Rozendaal et al., 2011), this makes it less likely that children will defend themselves to its persuasive appeals. In such a relatively low elaborate, automatic level of processing, food cues in media messages can lead to consumption behavior via simple and direct cue-reactivity mechanisms (Folkvord et al., 2016).

Moreover, the defining characteristic of these vlogs; that is, the social media influencers themselves, may trigger particularly powerful influence mechanisms that have so far only been associated with character marketing among young children (i.e., using animated characters on product packaging, see De Droog et al., 2012). It is conceivable that the mechanisms underlying the effectiveness of character marketing, such as parasocial relationship formation and identification with characters (De Droog et al., 2012), occur for social media influencers as well (Lee and Watkins, 2016). Similar to what animated characters represent for young children, social media influencers represent highly attractive characters with whom older children can build parasocial relationships, identify themselves, and whom they trust and believe (De Droog, 2012; Kapitan and Silvera, 2016; Lee and Watkins, 2016; Folkvord et al., 2019). Rather than a simple and direct cue-reactivity effect, these mechanisms involve longer term formation of positive associations with the advertised products, potentially leading to enduring attitudinal and behavioral change (Buijzen et al., 2010).

As yet, the relations between watching vlogs and unhealthy dietary patterns have not been investigated. Previous research that has focused on other types of food marketing has convincingly shown that exposure to media depicting unhealthy products, such as sugar-sweetened beverages and snacks, is associated with higher consumption of such products among children (e.g., Vereecken et al., 2006; Buijzen et al., 2008; Lipsky and Iannotti, 2012; Boyland et al., 2016; Folkvord et al., 2016; Pearson et al., 2018). These studies have generally studied dose-response associations, finding that the more children were exposed to the various forms of food marketing, the more they consumed the advertised products.

The question remains whether this is also the case for watching online vlogs. As argued above, it is conceivable that for vlogs the impact on unhealthy foods is even stronger than for traditional advertising. Therefore, the aim of the current Brief Research Report is to examine the impact of vlogs on children’s unhealthy dietary behaviors. Specifically, drawing on three waves of longitudinal survey data from a large-scale project on youth’s healthy lifestyles, we analyzed the longitudinal relations between 8- to 12-year-old children’s frequency of watching vlogs and their consumption of unhealthy beverages and snacks.

## Materials and Methods

We used longitudinal data from the *MyMovez* project (MyMovez, 2019), a large-scale data collection funded by the European Research Council (ERC), investigating youth’s social networks in combination with individual, psychosocial, and other environmental factors related to a healthy lifestyle (see Bevelander et al., 2018 for a detailed description). All primary and secondary schools that followed a regular education program were eligible for participation. Collection of the three waves of data used in the current study took place during the Springs of 2016 (T1), 2017 (T2), and 2018 (T3).

### Participants

A total of 953 primary and secondary school children with an initial age between 8 and 15 years participated in the *MyMovez* project. The sample used in the study reported here consisted of 453 primary school children. The initial age of the children ranged between 8 and 12 years (*M* = 10.05; *SD* = 0.97). Boys and girls were represented about equally: 52.5% were girls. Almost all children were of Dutch origin (>90%). The majority of the children had a normal weight (70.4%), 7.8% were underweight, 16.3% were overweight, and 5.3% were extremely overweight (i.e., obese).

### Procedure

The *MyMovez* project received ethical approval of the ERC’s Ethical review board (project nr. ERC-COG-617253) and the host institution’s Ethics Committee of Social Sciences (ECSW2014-100614-222). Children were recruited through their schools. After gaining permission to participate from the schools’ directors, active written consent was obtained from children’s caretakers as well as the children themselves. During the data collection waves children received the *MyMovez* “Wearable Lab” for seven consecutive calendar days, including the weekend. This Wearable Lab consisted of a smartphone with a pre-installed research application and an activity tracking bracelet (Bevelander et al., 2018). Via the smartphone application, children received daily questionnaires at random time points between 7:00 am and 7:30 pm, but not during school hours, except for school breaks. The smartphone-administered questionnaires assessed, among other measures, children’s frequency of watching vlogs and unhealthy dietary behaviors (i.e., consumption of sugar-sweetened beverages and high energy-dense snacks) that were used in the present study.

### Measures

#### Frequency of Watching Vlogs

Following paradigms of self-report measurement of media exposure, exposure to vlogs was operationalized as Frequency of Watching Vlogs^1^. In a systematic comparison of self-reported advertising exposure measures among 8- to 12-year-olds, Opree et al. (2014) demonstrated that a question regarding the frequency of watching a specific media channel provides a reliable and valid estimate of exposure to advertising on that channel. Therefore, to measure Frequency of Watching Vlogs, children were asked “*How often do you watch vlogs (video weblogs)? Think of videos from vloggers such as EnzoKnol or BeautyNezz”* They could respond on a 6-point scale ranging from 1 = *never* to 6 = *always* (cf. Opree et al., 2014). T1: range 1–6, *M* = 3.66, *SD* = 1.52; T2: range 1–6, *M* = 3.44, *SD* = 1.55; T3: range 1–6, *M* = 3.29, *SD* = 1.39.

#### Consumption of Unhealthy Beverages

To assess Unhealthy Beverages Consumption, children indicated on three different days (i.e., every other day during each data collection wave) how much sweetened fruit juice, lemonade (based on sugar syrup), soda, energy-, and sports drinks they had drunk the day before (Smit et al., 2016, 2018; Bevelander et al., 2018). Response options ranged from 0 = *zero glasses per day* to 7 = *seven or more glasses per day*. An illustration was used to instruct the children that “one glass” also meant one can, bottle, or package of approximately 200 ml. A total score for Unhealthy Beverages Consumption was constructed by averaging children’s reported consumption on the five items over the 3 days: T1: range 0–4.75, *M* = 0.69, *SD* = 0.62; T2: range 0–4.00, *M* = 0.68, *SD* = 0.72; T3: range 0–4.25, *M* = 0.62, *SD* = 0.64.

#### Consumption of Unhealthy Snacks

To assess Unhealthy Snacks Consumption, children indicated on three different days (i.e., every other day during each data wave) how many pieces (i.e., units) of small cookies, large cookies, wrapped cookies, chocolates or bonbons, chocolate bars, sweets or licorice, potato chips or salty snacks, and nuts or peanuts they had consumed the day before (Ocke et al., 1997; Bevelander et al., 2018). A more detailed description of the items can be found in the *MyMovez* protocol (Bevelander et al., 2018). Response categories ranged from 0 = *none* to 6 = *six or more*. Averaging children’s reported consumption on the eight items over the 3 days produced a total score for Unhealthy Snacks Consumption: T1: range 0–5.00, *M* = 0.65, *SD* = 0.55; T2: range 0–4.19, *M* = 0.71, *SD* = 0.70; T3: range 0–3.63, *M* = 0.59, *SD* = 0.69.

#### Control Variables

We included Body Mass Index (BMI) and Family Affluence measured at T1 to account for individual differences that might affect the relations between watching vlogs and dietary behaviors. BMI for each child was calculated using the formula: weight in kg/height in m. *z*-BMI was calculated and was representative of standards for Dutch children (range −2.69 to 3.21, *M* = 0.17, *SD* = 1.08; Schönbeck et al., 2011). Family Affluence was captured with the second version of the Family Affluence Scale (FAS II; Boyce et al., 2006) (range 1–13, *M* = 8.54, *SD* = 1.84).

## Results

### Strategy of Analysis

**FIGURE 1:**
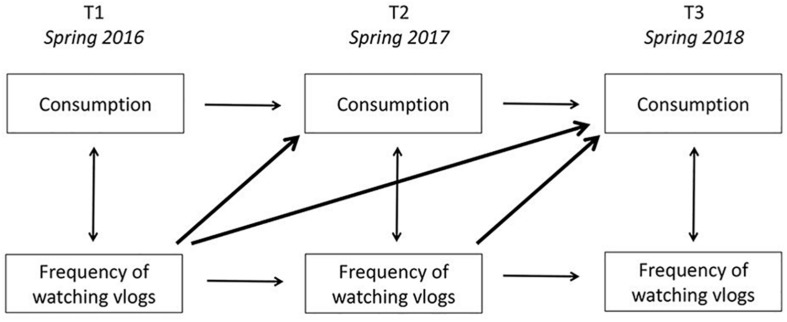
Analytical model.

The analyses consisted of two structural path models using Mplus Version 8.3, one for the influence of Frequency of Watching Vlogs on Unhealthy Beverages Consumption over time and one for the influence of Frequency of Watching Vlogs on Unhealthy Snacks Consumption over time. Figure 1 depicts the basic model tested in the analyses. The models included regression paths for the behaviors from T1 to T2 and T2 to T3 to account for interindividual stability in the behavior. In addition, the models controlled for BMI and Family Affluence at T1, and for cross-sectional covariances between Frequency of Watching Vlogs and Consumption at T1, T2, and T3. The parameters in the models were estimated applying the (full-information) maximum-likelihood estimator with robust standard errors (MLR in Mplus) to account for missing values and potential deviations from multivariate normality. The fit of the models was assessed with the following good fit indices: RMSEA (root mean square error of approximation, with a cut-off value of <0.08 and *p*-close >0.05), CFI (comparative fit index, with a cut-off value of >0.90), and χ^2^/df ratio, with a cut-off value of <3.0 (Hu and Bentler, 1999; Kline, 2011).

### Model Results

The model investigating Unhealthy Beverages Consumption showed a good fit to the observed data, RMSEA = 0.05, *p*-close = 0.591, CFI = 0.93, χ^2^/df = 1.88. Table 1 presents all regression paths estimated in the model examining Unhealthy Beverages Consumption. All stability paths were significant. Frequency of Watching Vlogs at T1 significantly predicted Unhealthy Beverages Consumption at T3. The other longitudinal regression paths were non-significant. This meant that when children (reported that they) watched more vlogs, they did not drink more unhealthy beverages in the subsequent year, but did drink more unhealthy beverages 2 years later.

**TABLE 1:** Results for the model predicting unhealthy beverages consumption.

	***b***	***SE***	***p***	**CI**
**Longitudinal regression paths**				
Watching vlogs T1 → Consumption T2	–0.03	0.08	0.658	[−0.18, 0.11]
Watching vlogs T2 → Consumption T3	–0.04	0.06	0.585	[−0.16, 0.09]
Watching vlogs T1 → Consumption T3	0.15	0.07	0.021	[0.02, 0.28]
**Stability regression paths**				
Consumption T1 → Consumption T2	0.51	0.05	<0.001	[0.40, 0.61]
Consumption T2 → Consumption T3	0.51	0.08	<0.001	[0.36, 0.66]
Watching vlogs T1 → Watching vlogs T2	0.28	0.08	<0.001	[0.13, 0.44]
Watching vlogs T2 → Watching vlogs T3	0.48	0.06	<0.001	[0.37, 0.59]

*Model controlling for BMI and FAS at T1, and for cross-sectional covariances between Frequency of Watching Vlogs and Consumption at T1, T2, and T3.*

The model investigating Unhealthy Snacks Consumption also showed a good fit to the observed data, RMSEA = 0.04, *p*-close = 0.647, CFI = 0.90, χ^2^/df = 1.79 Table 2, presenting all regression paths estimated in the model, demonstrates that all stability paths were significant, but that none of the longitudinal regression paths leading from Frequency of Watching Vlogs to Unhealthy Snacks Consumption were significant.

**TABLE 2:** Results for the model predicting unhealthy snacks consumption.

	***b***	***SE***	***p***	**CI**
**Longitudinal regression paths**				
Watching vlogs T1 → Consumption T2	–0.05	0.08	0.525	[−0.20, 0.10]
Watching vlogs T2 → Consumption T3	–0.09	0.14	0.534	[−0.35, 0.18]
Watching vlogs T1 → Consumption T3	0.10	0.10	0.330	[−0.10, 0.29]
**Stability regression paths**				
Consumption T1 → Consumption T2	0.45	0.09	<0.001	[0.27, 0.63]
Consumption T2 → Consumption T3	0.58	0.11	<0.001	[0.36, 0.80]
Watching vlogs T1 → Watching vlogs T2	0.29	0.08	<0.001	[0.13, 0.44]
Watching vlogs T2 → Watching vlogs T3	0.47	0.06	<0.001	[0.36, 0.59]

*Model controlling for BMI and FAS at T1, and for cross-sectional covariances between Frequency of Watching Vlogs and Consumption at T1, T2, and T3.*

## Discussion

This Brief Research Report presented the first study to investigate the longitudinal impact of watching vlogs on children’s dietary behaviors. Structural path modeling analyses of three waves of data showed that children’s self-reported frequency of watching vlogs was related to consumption of unhealthy beverages 2 years later. The analyses did not yield significant relations for beverages over a 1 year period, nor for snacks consumption over a one or 2-year period.

The effect size of the observed longitudinal relation between children’s frequency of watching vlogs and their consumption of unhealthy beverages can be interpreted as small (Maher et al., 2013), which is comparable to previous findings regarding more traditional types of food marketing (Vereecken et al., 2006; Buijzen et al., 2008; Lipsky and Iannotti, 2012; Boyland et al., 2016; Folkvord et al., 2016; Pearson et al., 2018). We had expected to find a stronger association, given the defining characteristics of social media influencers that should strengthen the impact of advertising. A potential explanation might lie in the great number of influencers and vlogs, and the large variety of product categories that they promote, also including for example beauty and clothing brands. It is likely that more specific insight into the food-related content of the vlogs that children are exposed to will yield more pronounced associations.

Moreover, the results were not consistent with regard to the short- and long-term relations modeled. There are several possible explanations why the analyses for beverages yielded an effect on the long term (2 years; from T1–T3), and not for the shorter term (1 year; from T1–T2, and T2–T3). First, the longer term relation might point toward a particular effect mechanism. The observed patterns are not in line with cue-reactivity explanation of advertising effects, which assumes that exposure to food cues directly and immediately evokes behavioral responses (cf. Folkvord et al., 2016). Even though there is convincing evidence for such a cue-response mechanism for other types of integrated advertising, the patterns observed for influencers in the current study suggest a more complex effect mechanism that needs more time to evolve. Given the specific features of influencer marketing, it is plausible that the mechanisms observed for character marketing among younger children (De Droog et al., 2011, 2012) also hold for influencer marketing.

To come to decisive conclusions about the mechanisms involved in the effects of social media influencer food marketing on unhealthy dietary behaviors, there is a need for further research. Specifically, there is a need for experimental research systematically varying the characteristics that are expected to increase the impact, including message factors (e.g., level of integration of the food promotion; Buijzen et al., 2010) and characteristics of the child-influencer relationship (e.g., level of identification; Folkvord et al., 2019). Vlogs with social media influencers may provide excellent stimulus materials for such experimental designs, with one recent randomized controlled trial providing a promising first step (Van Woudenberg et al., 2019). Comparing the effects of personally familiar with non-familiar vloggers on responses to the vlogs and physical activity behaviors, Van Woudenberg et al.’s findings showed that familiarity of the influencer improved adolescents’ viewing time of and attitudes toward the vlogs and vloggers.

Finally, an additional explanation for only observing a longer-term relation might also be found in the context and timing of the study. At the onset of the study, vlogs were still a relatively new phenomenon. It is conceivable that children who watched vlogs in those early days were less aware of this new form of embedded food marketing. Since then, knowledge may have accumulated, for example due to school-based media literacy programs that started to pay attention to the phenomenon. Moreover, at the time of the study a self-regulatory code for influencers was being developed in the Netherlands, which also received some media attention. These changing circumstances may have resulted in children becoming more aware of and critical about food marketing in vlogs.

## Conclusion

In conclusion, our findings have implications for the academic and the public debate about non-traditional forms of food marketing. Theoretically, they extend the literature of advertising effects by examining a rapidly evolving form of media with an arguably strong impact on dietary consumption. Our findings also have implications for policies regulating new forms of food marketing to children. Previously observed associations between more traditional forms of food marketing and children’s dietary behaviors have led to regulations and restrictions of food marketing in most Western countries. Our findings on Unhealthy Beverages Consumption indicate a similar need for food marketing by social media influencers. Several countries have taken steps in this direction, such as for example “The Social Code: YouTube” in the Netherlands, which involves a self-regulatory code for social media influencers under supervision of the Dutch Media Authority^2^. However, regulating online video content and social media influencers raises fundamental challenges for media policy, which the existing traditional media-based regulatory systems are often not able to address.

## Data Availability Statement

Requests to access the dataset can be made via DANS EASY doi: 10.17026/dans-zz9-gn44.

## Ethics Statement

The studies involving human participants were reviewed and approved by Ethics Committee Social Science (ECSS) – Radboud University. Written informed consent to participate in this study was provided by the participants’ legal guardian/next of kin.

## Author Contributions

All authors contributed to the study design. Data collection was performed by LB, KB, CS, and TW. LB, CS, and MB performed the data analyses and interpretation. MB drafted and finalized the manuscript. All authors provided critical revisions and approved the final version of the manuscript for submission.

## Conflict of Interest

The authors declare that the research was conducted in the absence of any commercial or financial relationships that could be construed as a potential conflict of interest.

## Footnotes

1 https://mediaexposuremeasures.org/

2 www.desocialcode.nl
